# Abdominal obesity and its association with health-related quality of life in adults: a population-based study in five Chinese cities

**DOI:** 10.1186/1477-7525-12-100

**Published:** 2014-06-13

**Authors:** Shunquan Wu, Rui Wang, An Jiang, Yingying Ding, Meijing Wu, Xiuqiang Ma, Yanfang Zhao, Jia He

**Affiliations:** 1 Department of Health Statistics, Second Military Medical University, No. 800 of XiangYin Road, Shanghai, China; 2 Department of Medical Microbiology and Parasitology, Second Military Medical University, Shanghai, China

**Keywords:** Abdominal obesity, Waist circumference, Waist-to-hip ratio, Health-related quality of life

## Abstract

**Background:**

This study aimed to investigate the prevalence of abdominal obesity and its association with the health-related quality of life (HRQOL) in a randomly selected Chinese sample.

**Methods:**

A population-based sample of 3,600 residents aged 18–80 years was selected randomly from 5 Chinese cities. Demographic information, and waist and hip circumference measurements were obtained. The Mandarin version of the Short Form 36 Health Survey questionnaire (SF-36) was used to assess the HRQOL. Waist circumference (WC) and waist-to-hip ratio (WHR) were used as measures of abdominal obesity, and the prevalence of abdominal obesity and its association with HRQOL were analysed.

**Results:**

Among the 3,184 participants included in the analysis, the prevalence of abdominal obesity was about 45% in both women and men as evaluated by WC, and about 40% in women and 33% in men as evaluated by WHR. The prevalence varied by city, region, age, marital status, education level, family income, smoking, and the presence of chronic diseases. Both WC and WHR increased with age, and men had larger WC and WHR than women in most age groups. In women, abdominal obesity, as determined by both WC and WHR, was associated with meaningful impairments in 4 physical health scales and 2 mental health scales. In men, abdominal obesity, as determined by WC, was associated with 1 physical health scale and 1 mental health scale, and it was associated with 2 physical health scales based on WHR.

**Conclusions:**

Physical health, but not mental health, was more vulnerable to impairment with abdominal obesity, and the impairments varied between genders. Public health agencies should emphasize that abdominal obesity impairs physical health.

## Background

In recent years, the prevalence of obesity has increased dramatically in many countries. In 2009–2010, 35.7% of adults in the United States were obese [1]. In the past decades, China has undergone rapid economic development, which has resulted in the transition of dietary patterns to a diet characterized by high fat and high energy, as well as a more sedentary lifestyle. It was reported that the overall prevalence of obesity in China increased from 3.3% to 5.6% during the period of 1992–2002, an increase of 80.6% [2].

In clinical, research, and public health fields, body mass index (BMI) has been commonly used to define overall obesity. However, BMI does not consider the pattern of body fat distribution. Abdominal obesity, a key component of obesity, can be assessed using simple measures such as waist circumference (WC) or waist-to-hip ratio (WHR) [3-5], and it is a stronger predictor of obesity-related morbidity and mortality risk than overall obesity as assessed by BMI [6-8]. Some studies have recommended WC as a better indicator of abdominal obesity and a better predictor of disease risk than other anthropometric measurements [4,9,10]. According to Esmaillzadeh et al., WHR was a better predictor of cardiovascular risk than BMI and WC in Iranian adult men [11].

Although abdominal obesity has been extensively studied, much of the research has focused on specific age groups, sex groups, or patient groups [4,9-13]. Early studies indicated that overweight subjects are more likely to have poorer physical and emotional functioning status [14,15], measures of the health-related quality of life (HRQOL), and it is important in terms of public health. To our knowledge, there are few studies reporting the relationship between adverse fat distribution (i.e., large WC and high WHR) and HRQOL, particularly in the general population. A previous study has shown that a high WC is more likely to be associated with impaired quality of life and disability, negatively affecting basic activities of daily life in a Dutch population [16]. As WC and WHR distributions differ in the Chinese population, the influence of abdominal obesity on HRQOL may be different from that in Western countries. The purpose of this study was to investigate the prevalence of abdominal obesity and evaluate the influence of abdominal obesity on HRQOL in adults among the general population in 5 Chinese cities.

## Methods

### Study design and sample

The data for this study were derived from our previous epidemiology survey on gastrointestinal diseases in 5 Chinese cities, including Shanghai, Beijing, Wuhan, Xian, and Guangzhou. A detailed description of the study population and the methods of the survey have been previously published [17]. Briefly, the survey was conducted among 3,600 residents aged 18–80 years from April 2007 to January 2008. The subjects were sampled by a randomized stratified multiple-stage method from the 5 cities, with the age and gender distribution in accordance with the distribution of the local population based on government-published population census statistics. Basic demographic information of the residents was obtained from local residential committee offices prior to conducting the survey. We excluded residents who were illiterate, not in the 18–80 age group, or suffering from psychiatric illnesses or other disabilities. Of the 3,600 sampled residents, 3,219 agreed to be interviewed, a response rate of 89.4%. Data from 5 participants were excluded because of logical errors or insufficient completion, and we also excluded 30 participants who did not receive a physical examination. As a result, analyses were conducted on 3,184 respondents. All respondents gave written informed consent before participation. The study was approved by the Second Military Medical University Ethics Committee in Shanghai, China.

Questionnaires were self-completed in the local residential committee offices, with trained interviewers providing an explanation for any unclear questions. Demographic information was collected, including residential region, sex, age, marital status, education level, current occupation, family income, smoking behaviour, drinking behaviour, and frequency of physical activity. In the questionnaire, the region of residence was classified into 2 categories (urban area and rural area); marital status was classified into 3 categories (married, unmarried, and separated/divorced/widowed). Education level was classified into 3 categories (primary school/uneducated, secondary/high school, and college graduate), and current occupation was classified into 7 categories (government employees, professional or technician, agricultural or fisheries workers, blue-collar workers, personal services, current students, and personnel). Family income was classified into 3 categories (<1,999 yuan/month, 2,000–4,999 yuan/month, and ≥5,000 yuan/month). Smoking behaviour was classified into 7 categories (never smoked, 1–5 cigarettes/day, 6–10 cigarettes/day, 11–15 cigarettes/day, 16–20 cigarettes/day, ≥21 cigarettes/day, and former smoker), and drinking behaviour was classified into 4 categories (never drink alcohol, <4 times/month, ≥1 time/week, and ≥1 time/day). The frequency of physical activity was classified into 4 categories (never, <4 times/month, ≥1 time/week, and ≥1 time/day). Respondents were also asked whether they had been diagnosed with chronic diseases, including hypertension, angina pectoris, cerebrovascular disease, chronic bronchitis, rheumatoid arthritis, osteoarthritis, or diabetes. A physical examination was conducted for each respondent to measure weight, height, and waist and hip circumference.

### WC and WHR

WC and WHR were used to evaluate abdominal obesity. WC was measured during minimal respiration at the mid-point between the lowest rib and the iliac crest to the nearest 0.1 cm, and hip circumference was measured at the point of maximum buttock extension to the nearest 0.1 cm. WHR was calculated as WC divided by hip circumference. All measurements were taken by trained investigators.

As different populations may have different optimal cutoff points for anthropometric measurements in determining obesity, we used cutoff points for WC and WHR that have previously suggested specifically for the Chinese population [18-20]. The WC categories are as follows: (i) normal WC, <80 cm for women and <85 cm for men; (ii) mild abdominal obesity, 80–90 cm for women and 85–95 cm for men; and (iii) severe abdominal obesity, ≥90 cm for women and ≥95 cm for men. The WHR categories are as follows: (i) normal WHR, <0.85 for women and <0.90 for men; and (ii) abdominal obesity, ≥0.85 for women and ≥0.90 for men.

### Measures of health-related quality of life

We assessed the respondents’ HRQOL by using the Mandarin version of the Short Form 36 Health Survey questionnaire (SF-36) that was translated from the International Quality of Life Assessment (IQOLA) SF-36 Standard UK Version 1.0 by experts at Zhejiang University; the SF-36 is a generic HRQOL instrument that has been tested for its reliability and validity [21,22]. The questionnaire has proved useful in monitoring population health, estimating the burden of different diseases, monitoring outcomes in clinical practice, and evaluating treatment effects. It comprises 36 questions describing 8 dimensions: physical functioning (PF), role limitations due to physical problems (RP), bodily pain (BP), general health perception (GH), vitality (VT), social functioning (SF), role limitations due to emotional problems (RE), and mental health (MH). The SF-36 dimensions can also be divided into 2 categories: a Physical Component Summary (PCS) and a Mental Component Summary (MCS), which represent physical function and wellbeing and emotional wellbeing, respectively.

The responses in the SF-36 were constructed by the Likert method of summated ratings. The raw score of each of the 8 dimensions was derived by summing the item scores and converting it to a value for the dimension from 0 (worst possible health state measured by the questionnaire) to 100 (best possible health state). The PCS and MCS scores were calculated using standard scoring algorithms [23].

### Statistical analysis

The data were analysed using Statistical Analysis System (SAS) 9.1.3. All hypothesis tests used two-sided tests, and *p*-values of less than 0.05 were considered statistically significant. As the cut points of anthropometric measures were different between women and men, analyses were conducted separately for women and men. Differences of WC distribution and WHR distribution among different cities, regions, ages, marital status, education levels, occupations, family income levels, smoking behaviour, drinking behaviour, frequency of physical activity, and the presence of specific chronic diseases were analysed by the Cochran-Mantel-Haenszel test and the Chi-square test, respectively. Student’s t test was used to compare differences in WC and WHR between women and men. Student-Newman-Keuls-q (SNK-*q*) test, a statistical method for pairwise comparison between groups, was used to compare the differences of WC and WHR among different age groups. Differences of HRQOL among different WC categories and WHR categories were analysed using analysis of variance (ANOVA), with the SNK-*q* test used for multiple comparisons. There was a ceiling effect for HRQOL in this study, meaning that the majority of participants had high SF-36 scores (many of them had an SF-36 score of 100). Consequently, the relationship between WC and HRQOL, as well as between WHR and HRQOL, was further analysed by logistic regression, adjusted by region, gender, age, marital status, education level, current job, monthly family income, smoking, drinking, frequency of activity, and chronic disease, in which the dependent variable was suboptimal HRQOL (0 represented SF-36 scores equal to 100, and 1 represented SF-36 scale scores <100).

## Results

### Characteristics of participants and the prevalence of abdominal obesity

**Figure 1:**
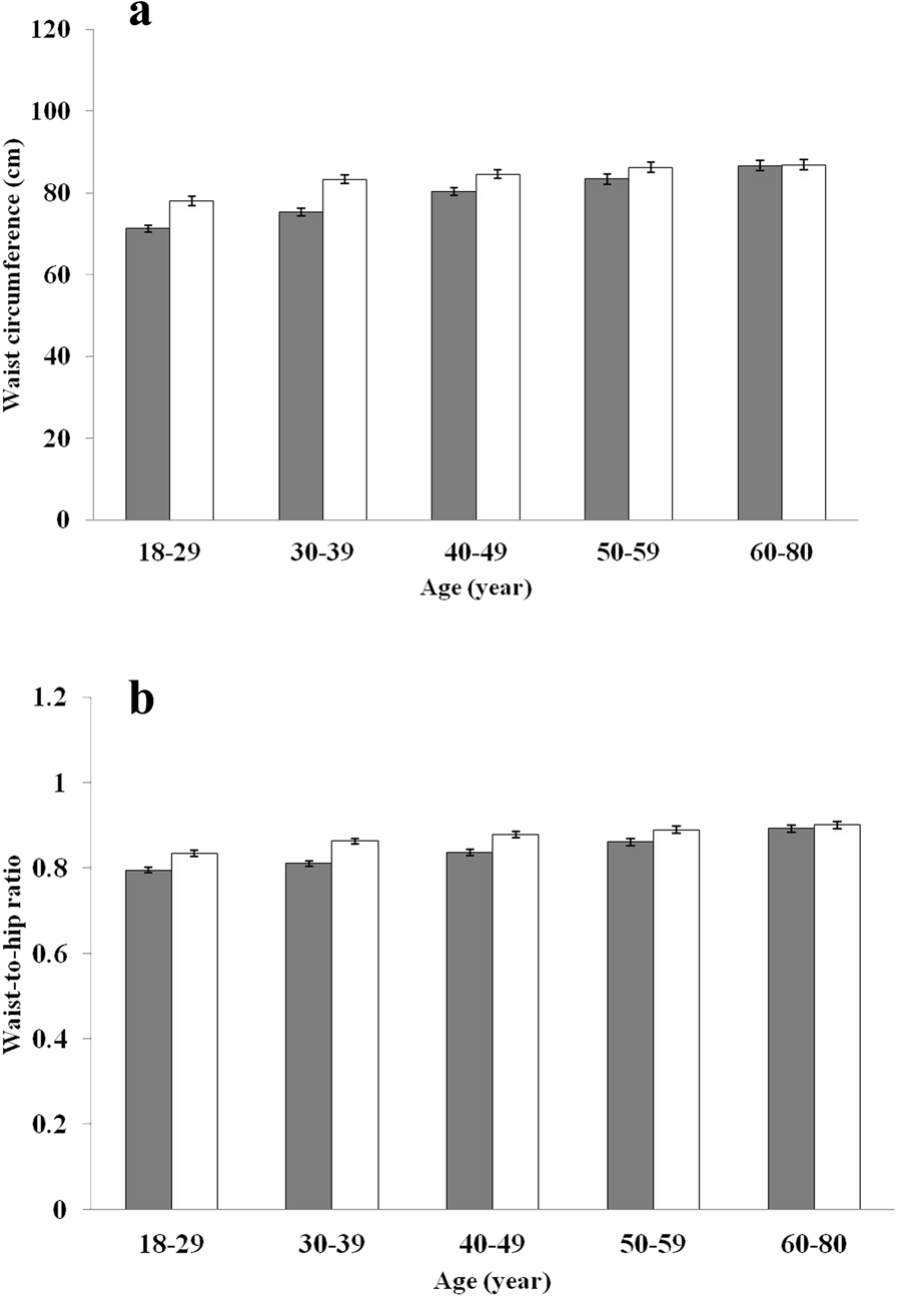
**Mean baseline waist circumference and waist-to-hip ratio by gender and age. a**. waist circumference; **b**. waist-to-hip ratio; Grey bars, women; white bars, men; error bars, the 95% CI.

The mean age of participants was 42.8 years for women (1,673 participants) and 42.2 years for men (1,511 participants). Mean WC and WHR were 78.6 cm and 0.83 for women, and 83.3 cm and 0.87 for men, respectively. WC and WHR differed by age and gender, as depicted in Figure 1. In the 60–80 year age group, mean WC and WHR were similar between genders; in the other 4 age groups, mean WC and WHR were significantly higher in men than in women. WC and WHR significantly increased with age.

As evaluated by WC categories, the prevalence of mild abdominal obesity and severe abdominal obesity were 27.1% and 17.0% for women, and 29.9% and 14.4% for men, respectively. The prevalence of abdominal obesity among women and men was 39.2% and 33.1%, respectively, as evaluated by WHR categories. Overall, the participants had very little missing demographic information (<1%), and we excluded subjects from the analysis if they had missing data on certain variables. As shown in Tables 1 and 2, according to WC categories, the prevalence of mild and severe abdominal obesity varied significantly by city, region, age, marital status, education level, family income and smoking situation among both women and men. Abdominal obesity was more frequent in women who engaged in manual work, women who never engaged in physical activity, women who engaged in physical activity at least once per day, and men who drank alcohol. Relative to those with normal WC, women with abdominal obesity tended to have chronic diseases, including hypertension, angina pectoris, cerebrovascular disease, chronic bronchitis, rheumatoid arthritis, osteoarthritis, and diabetes. Results were similar for men with the exception of rheumatoid arthritis and osteoarthritis. According to the WHR categories, the prevalence of abdominal obesity varied significantly by city, age, marital status, education level and frequency of physical activity among both women and men. Abdominal obesity was more frequent in women who lived in rural areas, women who engaged in manual work, women who had a lower level of family income (<2,000 yuan/month), and men who smoked and drank alcohol. All chronic diseases were more prevalent among participants with abdominal obesity than in those with normal WHR, with the exception of rheumatoid arthritis in men.

**Table 1:** Characteristics of women as categorized by WC and WHR

**N (%)**	**WC categories (cm)**^**a**^				**WHR categories**^**b**^		
	**Normal WC (<80)**	**Mild abdominal obesity (80–90)**	**Severe abdominal obesity (≥90)**	***P*-value**	**Normal WHR (<0.85)**	**Abdominal obesity (≥0.85)**	***P*-value**
Total	936(55.9)	453(27.1)	284(17.0)		1018(60.9)	655(39.2)	
City							
Shanghai	189(53.8)	111(31.6)	51(14.5)	<0.001	218(62.1)	133(37.9)	<0.001
Beijing	151(45.5)	96(28.9)	85(25.6)		160(48.2)	172(51.8)	
Wuhan	193(57.6)	85(25.4)	57(17.0)		207(61.8)	128(38.2)	
Xian	179(53.4)	90(26.9)	66(19.7)		210(62.7)	125(37.3)	
Guangzhou	224(70.0)	71(22.2)	25(7.8)		223(69.7)	97(30.3)	
Region							
Urban	479(58.8)	230(28.2)	106(13.0)	<0.001	546(67.0)	269(33.0)	<0.001
Rural	457(53.3)	223(26.0)	178(20.7)		472(55.0)	386(45.0)	
Age (years)							
18–29	322(84.5)	49(12.9)	10(2.6)	<0.001	307(80.6)	74(19.4)	<0.001
30–39	260(71.4)	81(22.3)	23(6.3)		277(76.1)	87(23.9)	
40–49	204(49.9)	132(32.3)	73(17.8)		263(64.3)	146(35.7)	
50–59	90(34.9)	95(36.8)	73(28.3)		106(41.1)	152(58.9)	
60–80	60(23.0)	96(36.8)	105(40.2)		65(24.9)	196(75.1)	
Marital status							
Married	687(52.3)	382(29.1)	244(18.6)	<0.001	777(59.2)	536(40.80)	<0.001
Unmarried	213(87.0)	28(11.4)	4(1.6)		202(82.4)	43(17.6)	
Separated/divorced/widowed	36(31.3)	43(37.4)	36(31.3)		39(33.9)	76(66.1)	
Education							
Primary school/uneducated	136(35.5)	125(32.6)	122(31.9)	<0.001	135(35.3)	248(64.7)	<0.001
Secondary/high school	578(58.0)	269(27.0)	149(15.0)		651(65.4)	345(34.6)	
College graduate	222(75.5)	59(20.1)	13(4.4)		232(78.9)	62(21.1)	
Occupation^c^							
Office worker	299(70.4)	89(20.9)	37(8.7)	<0.001	318(74.8)	107(25.2)	<0.001
Manual worker	635(51.0)	364(29.2)	247(19.8)		700(56.2)	546(43.8)	
Family income/month (Yuan)							
~1999	439(49.6)	251(28.3)	196(22.1)	<0.001	463(52.3)	423(47.7)	<0.001
2000~	406(62.3)	171(26.2)	75(11.5)		459(70.4)	193(29.6)	
5000~	87(66.9)	30(23.1)	13(10.0)		91(70.0)	39(30.0)	
Smoking^d^							
Non-smokers	918(56.2)	443(27.1)	272(16.7)	0.044	999(61.2)	634(38.8)	0.080
Smokers	18(45.0)	10(25.0)	12(30.0)		19(47.5)	21(52.5)	
Drinking^e^							
Non-drinkers	907(56.0)	436(27.0)	275(17.0)	0.796	982(60.7)	636(39.3)	0.477
Drinkers	29(52.7)	17(30.9)	9(16.4)		36(65.5)	19(34.5)	
Physical activity							
Never	175(58.3)	80(26.7)	45(15.0)	<0.001	178(59.3)	122(40.7)	<0.001
<4 times/month	99(68.8)	28(19.4)	17(11.8)		106(73.6)	38(26.4)	
≥1 time/week	155(64.3)	56(23.2)	30(12.5)		168(69.7)	73(30.3)	
≥1 time/day	505(51.3)	288(29.2)	192(19.5)		565(57.4)	420(42.6)	
Hypertension							
No	898(61.2)	383(26.1)	187(12.7)	<0.001	965(65.7)	503(34.3)	<0.001
Yes	38(18.5)	70(34.2)	97(47.3)		53(25.8)	152(74.2)	
Angina pectoris							
No	932(56.8)	443(27.0)	265(16.2)	<0.001	1013(61.8)	627(38.2)	<0.001
Yes	4(12.1)	10(30.3)	19(57.6)		5(15.2)	28(84.8)	
Cerebrovascular disease							
No	924(57.1)	429(26.5)	265(16.4)	<0.001	1005(62.1)	613(37.9)	<0.001
Yes	12(21.8)	24(43.6)	19(34.6)		13(23.6)	42(76.4)	
Chronic bronchitis							
No	909(56.8)	433(27.0)	259(16.2)	<0.001	988(61.7)	613(38.3)	<0.001
Yes	27(37.5)	20(27.8)	25(34.7)		30(41.7)	42(58.3)	
Rheumatoid arthritis							
No	906(57.3)	428(27.0)	248(15.7)	<0.001	982(62.1)	600(37.9)	<0.001
Yes	30(33.0)	25(27.5)	36(39.5)		36(39.6)	55(60.4)	
Osteoarthritis							
No	909(56.9)	427(26.7)	261(16.3)	<0.001	989(61.9)	608(38.1)	<0.001
Yes	27(35.5)	26(34.2)	23(30.3)		29(38.2)	47(61.8)	
Diabetes							
No	929(57.4)	438(27.0)	252(15.6)	<0.001	1007(62.2)	612(37.8)	<0.001
Yes	7(12.9)	15(27.8)	32(59.3)		11(20.4)	43(79.6)	

WC, waist circumference.

WHR, waist-to-hip ratio.

^a^Cochran–Mantel–Haenszel test was used.

^b^Chi-square test was used.

^c^Office worker: individuals who engaged in one of the following occupations—government employees, professional or technician, students in school, and personnel. Manual worker: individuals who engaged in one of the following occupations—agricultural or fisheries workers, blue-collar worker, or personal services.

^d^Non-smoker: individuals who had never smoked or had stopped smoking. Smoker: individuals who smoked at least 1 cigarette per day.

^e^Non-drinker: individuals who did not drink alcohol. Drinker: individuals who drank at least 1 time per month.

**Table 2:** Characteristics of men as categorized by WC and WHR

**N (%)**	**WC categories (cm)**^**a**^				**WHR categories**^**b**^		
	**Normal WC (<85)**	**Mild abdominal obesity (85–95)**	**Severe abdominal obesity (≥95)**	***P*-value**	**Normal WHR (<0.90)**	**Abdominal obesity (≥0.90)**	***P*-value**
Total	842(55.7)	452(29.9)	217(14.4)		1011(66.9)	500(33.1)	
City							
Shanghai	119(41.0)	111(38.3)	60(20.7)	<0.001	163(56.2)	127(43.8)	<0.001
Beijing	96(37.4)	108(42.0)	53(20.6)		153(59.5)	104(40.5)	
Wuhan	208(64.0)	70(21.5)	47(14.5)		244(75.1)	81(24.9)	
Xian	198(64.1)	71(23.0)	40(12.9)		213(68.9)	96(31.1)	
Guangzhou	221(67.0)	92(27.9)	17(5.1)		238(72.1)	92(27.9)	
Region							
Urban	374(49.3)	255(33.6)	130(17.1)	<0.001	509(67.1)	250(32.9)	0.899
Rural	468(62.2)	197(26.2)	87(11.6)		502(66.8)	250(33.2)	
Age (years)							
18–29	264(75.0)	55(15.6)	33(9.4)	<0.001	295(83.8)	57(16.2)	<0.001
30–39	214(58.6)	106(29.0)	45(12.3)		270(74.0)	95(26.0)	
40–49	180(52.2)	113(32.7)	52(15.1)		219(63.5)	126(36.5)	
50–59	101(44.9)	83(36.9)	41(18.2)		122(54.2)	103(45.8)	
60–80	83(37.1)	95(42.4)	46(20.5)		105(46.9)	119(53.1)	
Marital status							
Married	590(51.0)	385(33.3)	181(15.7)	<0.001	724(62.6)	432(37.4)	<0.001
Unmarried	235(74.4)	52(16.5)	29(9.2)		264(83.5)	52(16.5)	
Separated/divorced/widowed	16(42.1)	15(39.5)	7(18.4)		22(57.9)	16(42.1)	
Education							
Primary school/uneducated	102(54.5)	60(32.1)	25(13.4)	0.021	109(58.3)	78(41.7)	0.026
Secondary/high school	575(58.6)	277(28.2)	130(13.2)		666(67.8)	316(32.2)	
College graduate	165(48.3)	115(33.6)	62(18.1)		236(69.0)	106(31.0)	
Occupation^c^							
Office worker	249(52.0)	158(33.0)	72(15.0)	0.105	317(66.2)	162(33.8)	0.690
Manual worker	591(57.5)	293(28.5)	144(14.0)		691(67.2)	337(32.8)	
Family income/month (Yuan)							
~1999	457(61.8)	185(25.0)	98(13.2)	<0.001	500(67.6)	240(32.4)	0.801
2000~	316(50.9)	208(33.5)	97(15.6)		414(66.7)	207(33.3)	
5000~	66(45.5)	58(40.0)	21(14.5)		94(64.8)	51(35.2)	
Smoking^d^							
Non-smokers	350(58.9)	170(28.6)	74(12.5)	0.028	426(71.7)	168(28.3)	0.001
Smokers	492(53.6)	282(30.8)	143(15.6)		585(63.8)	332(36.2)	
Drinking^e^							
Non-drinkers	531(59.7)	242(27.2)	117(13.1)	0.001	629(70.7)	261(29.3)	<0.001
Drinkers	311(50.2)	209(33.7)	100(16.1)		381(61.5)	239(38.5)	
Physical activity							
Never	115(50.9)	70(31.0)	41(18.1)	0.078	132(58.4)	94(41.6)	0.007
<4 times/month	87(54.4)	51(31.9)	22(13.8)		115(71.9)	45(28.1)	
≥1 time/week	139(58.6)	64(27.0)	34(14.4)		171(72.1)	66(27.9)	
≥1 time/day	499(56.6)	265(30.0)	118(13.4)		591(67.0)	291(33.0)	
Hypertension							
No	784(60.3)	364(28.0)	152(11.7)	<0.001	916(70.5)	384(29.5)	<0.001
Yes	58(27.5)	88(41.7)	65(30.8)		95(45.0)	116(55.0)	
Angina pectoris							
No	836(56.1)	440(29.6)	213(14.3)	0.036	1001(67.2)	488(32.8)	0.031
Yes	6(27.3)	12(54.5)	4(18.2)		10(45.5)	12(54.5)	
Cerebrovascular disease							
No	830(56.3)	436(29.6)	208(14.1)	0.005	992(67.3)	482(32.7)	0.042
Yes	12(32.4)	16(43.3)	9(24.3)		19(51.4)	18(48.6)	
Chronic bronchitis							
No	809(56.3)	427(29.7)	201(14.0)	0.026	972(67.6)	465(32.4)	0.008
Yes	33(44.6)	25(33.8)	16(21.6)		39(52.7)	35(47.3)	
Rheumatoid arthritis							
No	808(55.7)	433(29.8)	210(14.5)	0.693	974(67.1)	477(32.9)	0.379
Yes	34(56.7)	19(31.7)	7(11.7)		37(61.7)	23(38.3)	
Osteoarthritis							
No	826(56.0)	437(29.6)	213(14.4)	0.561	993(67.3)	483(32.7)	0.049
Yes	16(45.7)	15(42.9)	4(11.4)		18(51.4)	17(48.6)	
Diabetes							
No	832(56.9)	426(29.1)	205(14.0)	<0.001	990(67.7)	473(32.3)	<0.001
Yes	10(20.8)	26(54.2)	12(25.0)		21(43.8)	27(56.2)	

WC, waist circumference.

WHR, waist-to-hip ratio.

^a^Cochran–Mantel–Haenszel test was used.

^b^Chi-square test was used.

^c^Office worker: individuals who engaged in one of the following occupations—government employees, professional or technician, students in school, and personnel. Manual worker: individuals who engaged in one of the following occupations—agricultural or fisheries workers, blue-collar worker, or personal services.

^d^Non-smoker: individuals who had never smoked or had stopped smoking. Smoker: individuals who smoked at least 1 cigarette per day.

^e^Non-drinker: individuals who did not drink alcohol. Drinker: individuals who drank at least 1 time per month.

### The relationship between abdominal obesity and HRQOL

There was a gender-dependent magnitude between abdominal obesity and HRQOL, as shown in Table 3. In women, participants with normal WC or normal WHR had significantly higher HRQOL than those with abdominal obesity in the majority of the survey scales. However, among men, participants with abdominal obesity had a significantly lower HRQOL than those with normal WC or WHR in fewer survey scales.

**Table 3:** Mean SF-36 scale and summary scores (standard deviation) by WC, WHR, and gender

**Scales**	**WC categories**			**WHR categories**	
	**Normal WC**	**Mild abdominal obesity**	**Severe abdominal obesity**	**Normal WHR**	**Abdominal obesity**
Women					
Physical functioning (PF)	95.5(9.2)	91.6(14.3)^a^	84.4(21.0)^a, b^	95.3(9.2)	88.3(18.2)^c^
Role-physical (RP)	90.9(25.2)	86.2(31.2)^a^	75.2(40.2)^a, b^	90.1(26.6)	82.1(35.1)^c^
Bodily pain (BP)	88.8(17.3)	86.0(21.0)^a^	81.2(24.2)^a, b^	88.1(18.0)	84.7(22.3)^c^
General health (GH)	70.3(20.6)	69.9(21.8)	61.3(23.4)^a, b^	70.2(20.6)	65.8(23.0)^c^
Vitality (VT)	69.0(17.8)	67.2(20.3)	61.8(21.8)^a, b^	68.5(18.2)	65.4(20.9)^c^
Social functioning (SF)	88.6(15.1)	87.6(16.1)	84.7(17.4)^a, b^	88.7(15.0)	86.1(17.0)^c^
Role-emotional (RE)	88.6(28.3)	89.3(28.6)	84.6(34.6)	89.2(27.8)	86.61(32.0)
Mental health (MH)	77.7(15.1)	78.1(16.5)	75.7(17.5)	77.4(15.4)	77.5(16.7)
Physical component summary (PCS)	53.9(5.7)	52.1(7.65)^a^	48.4(9.4)^a, b^	53.7(5.9)	50.7(8.7)^c^
Mental component summary (MCS)	51.2(7.8)	51.8(8.1)	51.0(8.9)	51.3(7.9)	51.5(8.4)
Men					
Physical functioning (PF)	96.2(9.1)	95.7(9.5)	92.8(16.0)^a, b^	96.2(9.4)	94.3(12.4)^c^
Role-physical (RP)	91.7(25.2)	90.3(25.9)	87.3(30.7)	91.7(24.9)	88.5(28.9)^c^
Bodily pain (BP)	90.0(17.6)	89.5(18.4)	88.6(19.0)	90.0(17.6)	89.0(18.9)
General health (GH)	71.2(20.2)	70.7(20.2)	71.7(19.2)	71.5(20.1)	70.5(20.0)
Vitality (VT)	70.6(17.5)	70.6(18.9)	70.9(18.3)	71.9(17.2)	70.0(19.5)
Social functioning (SF)	88.9(15.8)	88.9(15.8)	84.9(18.5)^a, b^	88.8(16.0)	87.3(16.8)
Role-emotional (RE)	90.9(26.4)	91.7(25.3)	90.3(27.5)	91.2(25.8)	90.8(26.9)
Mental health (MH)	76.7(15.9)	79.9(15.2)^a^	78.1(16.2)^a^	77.3(15.7)	78.9(16.0)
Physical component summary (PCS)	54.4(6.2)	53.6(6.3)	53.1(8.0)^a, b^	54.3(6.2)	53.3(7.1)^c^
Mental component summary (MCS)	51.3(8.0)	52.4(7.5)	51.7(8.0)	51.6(7.7)	52.0(8.2)

WC, waist circumference.

WHR, waist-to-hip ratio.

^a^*P* < 0.05, compared to the normal WC group by SNK-q test.

^b^*P* < 0.05, compared to the mild abdominal obesity group by SNK-q test.

^c^*P* < 0.05, compared to the normal WHR group by analysis of variance.

After further consideration of lifestyle variables, socio-demographic variables, and chronic diseases, we found that women with severe abdominal obesity had a lower HRQOL in RP scale based on WC categories, and women with mild abdominal obesity had a higher HRQOL in the VT scale. In men, only SF had a lower score in those with severe abdominal obesity, and those with mild abdominal obesity had a higher HRQOL in the PF scale (Table 4). Based on WHR categories, similar HRQOL were found in all survey scales in both women and men.

**Table 4:** **Associations between abdominal obesity and HRQOL from logistic regression analysis [OR (95% CI)]**^**a**^

**Scales**	**WC categories (cm)**^**b**^		**WHR**^**c**^
	**Mild abdominal obesity**	**Severe abdominal obesity**	**Abdominal obesity**
Women			
Physical functioning (PF)	1.04(0.80, 1.36)	1.40(0.99, 1.98)	1.07(0.83, 1.36)
Role-physical (RP)	1.11(0.80, 1.54)	**1.56(1.06, 2.29)**	1.09(0.81, 1.47)
Bodily pain (BP)	0.97(0.75, 1.25)	1.05(0.76, 1.45)	0.86(0.68, 1.09)
General health (GH)	1.26(0.63, 2.55)	2.23(0.62, 8.05)	0.73(0.40, 1.36)
Vitality (VT)	**0.45(0.22, 0.90)**	0.74(0.27, 2.05)	0.52(0.27, 1.00)
Social functioning (SF)	1.13(0.89, 1.45)	1.37(0.99, 1.88)	1.12(0.89, 1.42)
Role-emotional (RE)	0.80(0.56, 1.14)	0.78(0.51, 1.20)	0.93(0.67, 1.28)
Mental health (MH)	0.66(0.40, 1.08)	1.40(0.65, 3.01)	0.69(0.43, 1.12)
Men			
Physical functioning (PF)	**0.73(0.55, 0.97)**	0.73(0.51, 1.06)	0.89(0.69, 1.16)
Role-physical (RP)	1.29(0.89, 1.87)	1.37(0.86, 2.18)	1.14(0.81, 1.61)
Bodily pain (BP)	0.91(0.70, 1.20)	0.84(0.59, 1.20)	0.94(0.73, 1.21)
General health (GH)	0.95(0.48, 1.87)	0.61(0.26, 1.39)	1.48(0.76, 2.91)
Vitality (VT)	0.61(0.32, 1.18)	0.49(0.22, 1.11)	0.91(0.50, 1.65)
Social functioning (SF)	0.98(0.76, 1.26)	**1.41(1.02, 1.96)**	1.17(0.92, 1.47)
Role-emotional (RE)	0.99(0.67, 1.46)	1.13(0.69, 1.84)	1.05(0.73, 1.50)
Mental health (MH)	0.86(0.49, 1.50)	0.58(0.30, 1.12)	0.96(0.58, 1.58)

WC, waist circumference.

WHR, waist-to-hip ratio.

^a^The relationship between WC, WHR, and HRQOL was analysed by logistic regression, adjusted by region, gender, age, marital status, educational level, current job, monthly family income, smoking behaviour, drinking behaviour, frequency of activities, and chronic diseases, in which the dependent variable was suboptimal HRQOL (0 represents that SF-36 scale scores equal to 100, and 1 represents SF-36 scale scores <100).

^b^Mild abdominal obesity based on WC: 80–90 cm for women and 85–95 cm for men. Severe abdominal obesity based on WC: ≥90 cm for women and ≥95 cm for men.

^c^Abdominal obesity based on WHR: ≥0.85 for women and ≥0.90 for men.

Bold values indicate that the 95% CI does not include 1.

## Discussion

In the present study, the prevalence of abdominal obesity differed by some socio-demographic variables. The prevalence of abdominal obesity in women was higher in Beijing than in other cities; male participants from Shanghai and Beijing showed a higher prevalence of abdominal obesity. Shanghai and Beijing, as the economic centre and political centre of China, respectively, are more developed than other cities. The rhythm of life in these 2 cities is much faster, and people suffer from greater life and work stress. It has been proposed that stress reactions are linked with the development of abdominal obesity [24]. In addition, people with life and work stress are more likely to be smokers and drinkers [25,26]. As found in this and other studies [27,28], abdominal obesity is more frequently among people who smoked and drank alcohol. These could explain why participants in Shanghai and Beijing had higher proportions of abdominal obesity. For women, the prevalence of abdominal obesity was lower in urban areas than in rural areas, and it was opposite for men, as evaluated by WC categories. Abdominal obesity was more common among the elderly, particularly among women. Nearly 80% of the female participants had abdominal obesity after the age of 60 years. This is because older individuals are more likely to accumulate visceral fat than younger individuals [29]. Women who were separated, divorced, or widowed had a high proportion of abdominal obesity (nearly 70%). This can be partly explained by the fact that most women with these marital statuses were over the age of 60 years (65%). In addition, a low level of education, a lack of physical activity, and the presence of chronic diseases were associated with abdominal obesity. Similar results were found in a previous study, which investigated abdominal obesity in Iranian adults [30]. It is therefore imperative that public health officials target community health promotion to individuals with these specific risk factors.

A cross-sectional survey conducted in a nationally representative sample of Chinese adults in 2000–2001 showed that the mean WC and WHR were 77.2 cm and 0.83, respectively, for women and 79.6 cm and 0.86, respectively, for men [31]. Compared to the present study, there was an increase in the mean WC among Chinese adults since this time; however, there was no obvious change in the mean WHR. Men had higher mean WC and mean WHR than women before the age of 60 years, but this trend was not obvious after the age of 60 years. These results were similar to those found in South Korea, another East Asian country. The mean WC and mean WHR of adults in South Korea were 77.6 cm and 0.80 for women and 81.9 and 0.88 for men, respectively [32]. A national survey investigating trends in abdominal obesity among Korean adults found that after the age of 60 years, women had a higher mean WC than men in 1998 and 2007, although before the age of 60 years, men consistently had a higher mean WC than women in 1998, 2001, 2005 and 2007 [33]. This indicates a similar age and gender pattern of abdominal obesity between the 2 East Asian countries. However, the United States exhibits a different pattern of abdominal obesity, with an obviously higher mean WC in men than in women in all adult age groups, even after the age of 60 years [34]. As the sample in this study was selected in only five cities of China but not a national one, when we compared the results with those conducted in overall population of China or other countries, the differences in sampling may be responsible for the observed differences in data.

The present study shows that abdominal obesity influences some HRQOL scale scores in both women than in men. However, the influence of abdominal obesity on mental health was not as serious as that on physical health. In women, all of the 4 physical health scales were affected by abdominal obesity, but only 2 mental health scales were affected. In men, only 1 of the 4 mental health scales (SF) was affected based on WC; no mental health scale was affected based on WHR. Men with abdominal obesity had a higher HRQOL with respect to MH than those with normal WC. The summary scores based on both the WC and WHR categories also suggest that abdominal obesity impairs physical health rather than mental health. This phenomenon is supported by a previous study, which indicated that individuals with a high WC were more likely to have poor physical function that limited many basic activities of daily life; however, there was little evidence to suggest that individuals with a high WC displayed poor mental health or role limitations due to emotional problems [16]. After further considering lifestyle variables, socio-demographic variables, and the presence of chronic disease, only RP in women and SF in men were impaired by severe abdominal obesity, based on WC. This also suggests that the physical health of women is associated with abdominal obesity.

The strength of this study is that the data were collected from a random sample of adults in the general population rather than in a specific population based on age groups or diseases. However, there were still some limitations. The cross-sectional nature of the study does not allow us to make causal inferences, and whether the impaired HRQOL was caused by abdominal obesity is difficult to discern. In addition, as there is no clear gold standard for measuring abdominal obesity, we used WC and WHR, resulting in some inconsistencies between the 2 categories. Further studies are needed to determine the causal relationship between abdominal obesity and HRQOL and to define a proper gold standard for measuring abdominal obesity.

## Conclusion

In conclusion, we conducted a population-based study in 5 Chinese cities to investigate the prevalence of abdominal obesity among adults in the general population and the relationship between abdominal obesity and HRQOL. The prevalence of abdominal obesity of adults in China was about 45% in both women and men as evaluated by WC, and it was about 40% in women and 33% in men as evaluated by WHR. Both WC and WHR increased with age, and men had larger WC and WHR than women in most age groups. Impairments in physical health, but not mental health, were associated with abdominal obesity, but the specific impairments varied between genders.

## Competing interests

SQW, RW, AJ, YYD, MJW, XQM, and YFZ have no conflict of interests. JH has served as the director of the Department of Health Statistics, Second Military Medical University.

## Authors’ contributions

JH conceived of the study and supervised all aspects of its implementation. SQW, RW, and YYD assisted with the survey, completed the statistical analyses and led the writing of different versions of the manuscript. AJ, MJW and YFZ assisted with the study, and XQM assisted with the survey and data analyses. All authors contributed to conceptualize ideas, interpret findings, and review the drafts of the manuscript, and they approved the final manuscript.
